# Crystal structure of 1,4,8,11-tetra­methyl-1,4,8,11-tetra­azonia­cyclo­tetra­decane bis­(perchlorate) dichloride from synchrotron X-ray data

**DOI:** 10.1107/S2056989020001322

**Published:** 2020-02-11

**Authors:** Dohyun Moon, Jong-Ha Choi

**Affiliations:** a Beamline Department, Pohang Accelerator Laboratory, POSTECH, Pohang 37673, Republic of Korea; b Department of Chemistry, Andong National University, Andong 36729, Republic of Korea

**Keywords:** crystal structure, 1,4,8,11-tetra­methyl-1,4,8,11-tetra­azonia­cyclo­tetra­deca­ne, bis­(perchlorate) dichloride, hydrogen bonding, synchrotron radiation

## Abstract

The crystal structure of title salt, C_14_H_36_N_4_^4+^·2ClO_4_^−^·2Cl^−^, has been determined using synchrotron radiation at 220 K. The structure determination reveals that protonation has occurred at all four amine N atoms. The asymmetric unit contains one half-cation (completed by crystallographic inversion symmetry), one perchlorate anion and one chloride anion. A distortion of the perchlorate anion is due to its involvement in hydrogen-bonding inter­actions with the cations. The crystal structure is consolidated by inter­molecular hydrogen bonds involving the 1,4,8,11-tetra­methyl-1,4,8,11-tetra­azonia­cyclo­tetra­decane N—H and C—H groups as donor groups, and the O atoms of the perchlorate and chloride anion as acceptor groups, giving rise to a three-dimensional network.

## Abstract 2

In the title salt, [C_14_H_36_N_4_]^4+^·2ClO_4_^−^·2Cl^−^, the macrocyclic cations lie about an inversion center. In the crystal, N–H⋯Cl, C–H⋯Cl and C–H⋯O hydrogen bonds connect the cations and anions, forming a three-dimensional network.

## Chemical context   

Tetra­aza­macrocycle 1,4,8,11-tetra­methyl-1,4,8,11-tetra­aza­cyclo­tetra­decane (TMC, C_14_H_32_N_4_) is one of the most useful aza­macrocycles because of its ability to act as an effective metal-ion binding site and its basic properties. N-Substituted TMC is a basic amine that may form a dication, C_14_H_34_N_4_^2+^, or a tetra­cation, C_14_H_36_N_4_^4+^, in which the N—H bonds are generally active in hydrogen-bond formation. These di- or tetra­ammonium cations may be suitable for the removal of toxic heavy-metal ions. Because of a difference in the chirality of the secondary NH centers, the macrocyclic compounds can exhibit five conformations, *viz. trans*-I (*RSRS*), *trans*-II (*RSRR*), *trans*-III (*RRSS*), *trans*-IV (S*RRS*) and *trans*-V (*RRRR*) (Choi, 2009 ▸). Previously, the crystal structures for *trans*-[Ni(TMC)(H_2_O)_2_]Cl_2_·2H_2_O, [Ni(TMC)](O_3_SCF_3_) (Barefield *et al.*, 1986 ▸), [Cu(TMC)(H_2_O)](ClO_4_)_2_·H_2_O (Lee *et al.*, 1986 ▸), [Cu(TMC)](ClO_4_)_2_ (Maimon *et al.*, 2001 ▸), [Ag(TMC)](ClO_4_)_2_ (Po *et al.*, 1991 ▸), [Cu(NCS)(TMC)]ClO_4_ (Lu *et al.*, 1998 ▸) and [Cu(TMC)](BF_4_)_2_ (Bucher *et al.*, 2001*b* ▸) have been characterized crystallographically. In addition, first-row transition-metal complexes of the form [*M* ^II^Cl(TMC)]^+^ [*M* = Zn (Alcock *et al.*, 1978 ▸), Mn (Bucher *et al.*, 2001*a* ▸), Ni (Nishigaki *et al.*, 2010 ▸), Fe (Bedford *et al.*, 2016 ▸) and Co (Van Heuvelen *et al.*, 2017 ▸)] have been determined. Two independent ring conformations, *trans*-III and *trans*-IV, in the crystal structure of free TMC were also found (Willey *et al.*, 1994 ▸), but there is no report of a structure with any combination of the 1,4,8,11-tetra­methyl-1,4,8,11-tetra­azonia­cyclo­tetra­decane cation and ClO_4_^−^ and Cl^−^ anions. We report here the preparation of a new compound [H_4_TMC](ClO_4_)_2_Cl_2_, (I), and its structural characterization by synchrotron single-crystal X-ray diffraction.
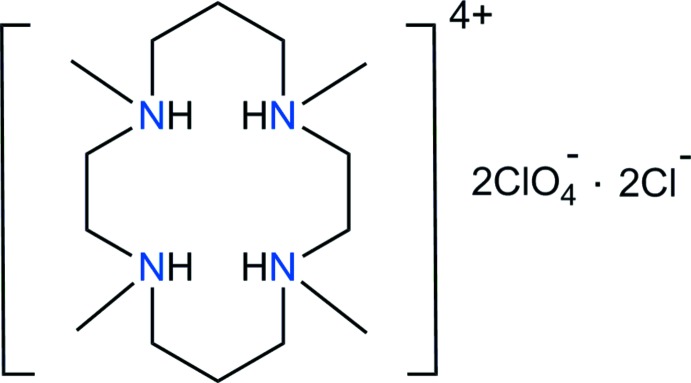


## Structural commentary   

**Figure 1:**
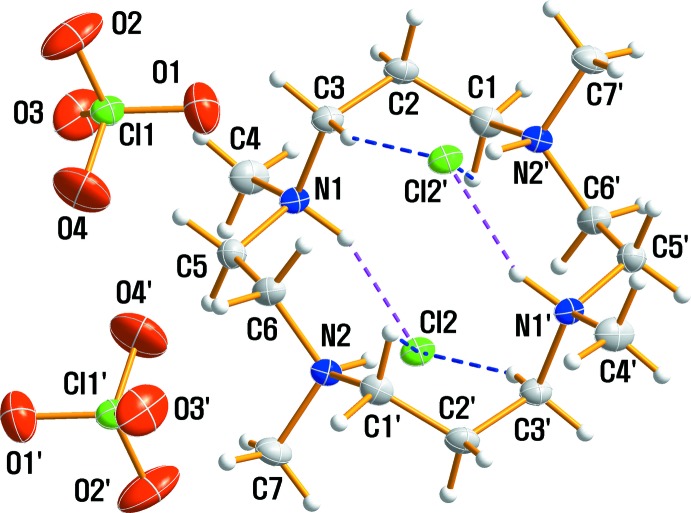
The structures of the mol­ecular components in (I), drawn with displacement ellipsoids at the 50% probability level. Dashed lines represent hydrogen-bonding inter­actions and primed atoms are related by the symmetry operation (−*x* + 1, −*y* + 1, −*z*).

An ellipsoid plot of the mol­ecular components in (I) is shown in Fig. 1 ▸ along with the atom-numbering scheme. The asymmetric unit consists of one half of the macrocycle, which lies about a center of inversion, one perchlorate anion and one chloride anion. The tetra-protonated amine of the title compound has a distorted *trans*-IV conformation, which is comparable to the *trans*-I or *trans*-III conformations of the dications in [H_2_TMC][As_4_O_2_Cl_10_] and [H_2_TMC][Sb_2_OCl_6_], respectively (Willey *et al.*, 1993 ▸). Within the centrosymmetric tetra-protonated C_14_H_36_N_4_^4+^ amine unit, the C—C and N—C bond lengths vary from 1.522 (2) to 1.527 (2) Å and from 1.5033 (19) to 1.5181 (18) Å, respectively. The N—C—C and C—N—C angles range from 113.55 (12) to 116.19 (12)° and 108.49 (12) to 112.37 (11)°, respectively. The bond lengths and angles within the tetra­ammonium cations are comparable to the corresponding values determined for the TMC moiety in [H_4_TMC]_2_[Sb_4_F_15_][HF_2_]F_4_ (Becker & Mattes, 1996 ▸), [H_2_TMC][As_4_O_2_Cl_10_], [H_2_TMC][Sb_2_OCl_6_] (Willey *et al.*, 1993 ▸), [H_4_TMC][H_2_TMC][W(CN)_8_]_2_·4H_2_O (Nowicka *et al.*, 2012 ▸), [Ga_2_(C_3_H_7_)_4_(OH)_2_](TMC) (Boag *et al.*, 2000 ▸), TMC (Willey *et al.*, 1994 ▸), *trans*-[Ni(TMC)(H_2_O)_2_]Cl_2_·2H_2_O (Barefield *et al.*, 1986 ▸), *trans*-[Os(TMC)(O)_2_](PF_6_)_2_ (Kelly *et al.*, 1996 ▸), [Cu(TMC)(H_2_O)](ClO_4_)_2_·H_2_O (Lee *et al.*, 1986 ▸), [Cu(NCS)(TMC)]ClO_4_ (Lu *et al.*, 1998 ▸) and [Cu(TMC)](BF_4_)_2_ (Bucher *et al.*, 2001*b* ▸). The Cl—O bond distances in the tetra­hedral ClO_4_^−^ anion range from 1.4180 (17) to 1.4380 (16) Å and the O—Cl—O angles from 106.85 (14)–110.94 (12)°. A distortion of the ClO_4_^−^ anion undoubtedly results from its involvement in hydrogen-bonding inter­actions with the cations.

## Supra­molecular features   

**Figure 2:**
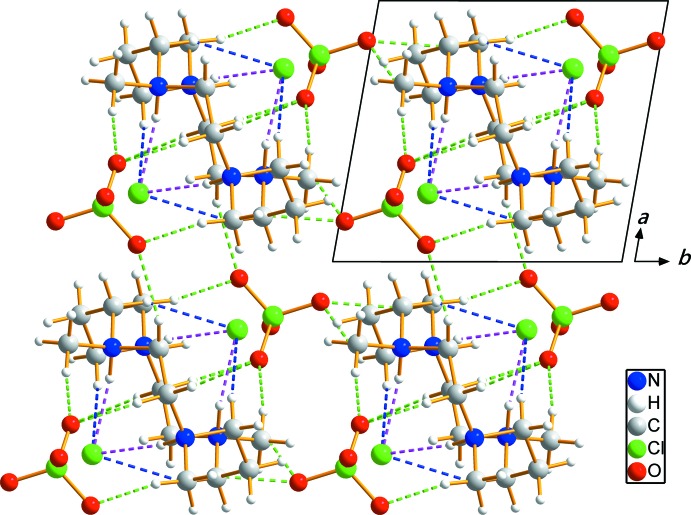
The crystal packing of compound (I), viewed perpendicular to the *ab* plane. Dashed lines represent N—H⋯Cl (purple), C—H⋯Cl (blue) and C—H⋯O (green) hydrogen-bonding inter­actions, respectively.

Extensive N—H⋯Cl, C—H⋯Cl and C—H⋯O hydrogen-bonding inter­actions occur in the crystal structure (Table 1 ▸). A crystal packing diagram of (I) viewed perpendicular to the *ab* plane is shown in Fig. 2 ▸.

The N—H⋯Cl and C—H⋯Cl hydrogen bonds link the two Cl^−^ anions to the C_14_H_36_N_4_^4+^ cation while C—H⋯O hydrogen bonds inter­connect neighboring cations with the ClO_4_^−^ anions. An extensive array of these contacts generates a three-dimensional network of mol­ecules, and these hydrogen-bonding inter­actions help to consolidate the crystal structure.

## Database survey   

A search of the Cambridge Structural Database (Version 5.41, November 2019; Groom *et al.*, 2016 ▸) gave just seven hits for organic compounds containing C_14_H_36_N_4_^4+^, C_14_H_34_N_4_^2+^ or C_14_H_32_N_4_ macrocycles: [C_14_H_36_N_4_]_2_[Sb_4_F_15_][HF_2_]F_4_ (Becker *et al.*, 1996 ▸), [C_14_H_34_N_4_][As_4_O_2_Cl_10_] and [C_14_H_34_N_4_][Sb_2_OCl_6_] (Willey *et al.*, 1993 ▸), [C_14_H_36_N_4_][C_14_H_34_N_4_][W(CN)_8_]_2_·4H_2_O (Nowicka *et al.*, 2012 ▸), [Ga_2_(C_3_H_7_)_4_(OH)_2_](C_14_H_32_N_4_) (Boag *et al.*, 2000 ▸) and (C_14_H_32_N_4_) (Willey *et al.*, 1994 ▸). However, the crystal structure of the title compound had not been deposited until now. The tetra-protonated amine of the title compound has a *trans*-IV conformation, which is comparable to the *trans*-I or *trans*-III conformation of the dications in [H_2_TMC][As_4_O_2_Cl_10_] and [H_2_TMC][Sb_2_OCl_6_], respectively (Willey *et al.*, 1993 ▸).

## Synthesis and crystallization   

The free ligand TMC (98%) was purchased from Sigma–Aldrich and used without further purification. All chemicals were reagent grade materials, and were used as received. TMC (0.128 g, 0.5 mmol) was dissolved in 15 mL of 6 *M* HCl, and 5 mL of a saturated solution of sodium perchlorate including chromium trioxide (0.1 g, 1 mmol) was added to the resulting solution at 298 K. The mixture was stirred for 2 h and the solution was filtered. Block-like pale yellow crystals of (I) suitable for X-ray structural analysis were unexpectedly obtained from the solution at 298 K over a period of a few days.

## Refinement   

Crystal data, data collection and structure refinement details are summarized in Table 2 ▸. All H atoms were placed in geometrically idealized positions and constrained to ride on their parent atoms, with C—H = 0.97–0.98 Å and N—H = 0.99 Å, and with *U*_iso_(H) values of 1.5 and 1.2 times the *U*_eq_ of the parent atoms.

## Supplementary Material

Crystal structure: contains datablock(s) I. DOI: 10.1107/S2056989020001322/vm2227sup1.cif

Structure factors: contains datablock(s) I. DOI: 10.1107/S2056989020001322/vm2227Isup2.hkl

Supporting information file. DOI: 10.1107/S2056989020001322/vm2227Isup3.cml

CCDC reference: 1980910

Additional supporting information:  crystallographic information; 3D view; checkCIF report

## Figures and Tables

**Table 1:** Hydrogen-bond geometry (Å, °)

*D*—H⋯*A*	*D*—H	H⋯*A*	*D*⋯*A*	*D*—H⋯*A*
N1—H1⋯Cl2	0.99	2.13	3.0701 (14)	159
N2—H2⋯Cl2	0.99	2.17	3.1038 (15)	156
C1—H1*A*⋯Cl2	0.98	2.77	3.6868 (17)	157
C5—H5*AB*⋯O3	0.98	2.39	3.351 (3)	167
C3—H3*A*⋯Cl2^i^	0.98	2.67	3.6274 (17)	164
C3—H3*AB*⋯O2^ii^	0.98	2.52	3.288 (3)	135
C4—H4*A*⋯O4^iii^	0.97	2.49	3.429 (3)	164
C4—H4*C*⋯O2^ii^	0.97	2.39	3.171 (3)	137
C5—H5*A*⋯O3^iii^	0.98	2.34	3.317 (3)	173
C6—H6*AB*⋯O4^iv^	0.98	2.31	3.231 (3)	156
C6—H6*A*⋯Cl2^i^	0.98	2.80	3.7414 (17)	161
C7—H7*B*⋯O3^iii^	0.97	2.40	3.333 (3)	161

Symmetry codes: (i) 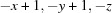; (ii) 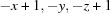; (iii) 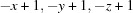; (iv) 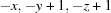.

**Table 2:** Experimental details

Crystal data	
Chemical formula	C_14_H_36_N_4_^4+^·2ClO_4_^−^·2Cl^−^
*M*_r_	530.27
Crystal system, space group	Triclinic, *P* 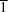
Temperature (K)	220
*a*, *b*, *c* (Å)	7.4990 (15), 8.0790 (16), 9.980 (2)
α, β, γ (°)	81.31 (3), 77.32 (3), 78.39 (3)
*V* (Å^3^)	574.2 (2)
*Z*	1
Radiation type	Synchrotron, λ = 0.610 Å
μ (mm^−1^)	0.36
Crystal size (mm)	0.10 × 0.10 × 0.08

Data collection	
Diffractometer	Rayonix MX225HS CCD area detector
Absorption correction	Empirical (using intensity measurements) (*HKL3000sm *SCALEPACK**; Otwinowski & Minor, 1997 ▸)
*T*_min_, *T*_max_	0.710, 1.000
No. of measured, independent and observed [*I* > 2σ(*I*)] reflections	6573, 3361, 3208
*R*_int_	0.038
(sin θ/λ)_max_ (Å^−1^)	0.706

Refinement	
*R*[*F* ^2^ > 2σ(*F* ^2^)], *wR*(*F* ^2^), *S*	0.052, 0.147, 1.05
No. of reflections	3361
No. of parameters	138
H-atom treatment	H-atom parameters constrained
Δρ_max_, Δρ_min_ (e Å^−3^)	0.97, −0.56

Computer programs: *PAL BL2D-SMDC Program* (Shin *et al.*, 2016 ▸), *HKL3000sm* (Otwinowski & Minor, 1997 ▸), *SHELXT2014* (Sheldrick, 2015*a* ▸), *SHELXL2018* (Sheldrick, 2015*b* ▸), *DIAMOND4* (Putz & Brandenburg, 2014 ▸) and *publCIF* (Westrip, 2010 ▸).

## supplementary crystallographic information

### Crystal data

**Table:**

C_14_H_36_N_4_^4+^·2ClO_4_^−^·2Cl^−^	*Z* = 1
*M**_r_* = 530.27	*F*(000) = 280
Triclinic, *P*1	*D*_x_ = 1.533 Mg m^−^^3^
*a* = 7.4990 (15) Å	Synchrotron radiation, λ = 0.610 Å
*b* = 8.0790 (16) Å	Cell parameters from 91694 reflections
*c* = 9.980 (2) Å	θ = 0.4–33.7°
α = 81.31 (3)°	µ = 0.36 mm^−^^1^
β = 77.32 (3)°	*T* = 220 K
γ = 78.39 (3)°	Block, pale yellow
*V* = 574.2 (2) Å^3^	0.10 × 0.10 × 0.08 mm

### Data collection

**Table:**

Rayonix MX225HS CCD area detector diffractometer	3208 reflections with *I* > 2σ(*I*)
Radiation source: PLSII 2D bending magnet	*R*_int_ = 0.038
ω scan	θ_max_ = 25.5°, θ_min_ = 1.8°
Absorption correction: empirical (using intensity measurements) (*HKL3000sm Scalepack*; Otwinowski & Minor, 1997)	*h* = −10→10
*T*_min_ = 0.710, *T*_max_ = 1.000	*k* = −11→11
6573 measured reflections	*l* = −14→14
3361 independent reflections	

### Refinement

**Table:**

Refinement on *F*^2^	0 restraints
Least-squares matrix: full	Hydrogen site location: inferred from neighbouring sites
*R*[*F*^2^ > 2σ(*F*^2^)] = 0.052	H-atom parameters constrained
*wR*(*F*^2^) = 0.147	*w* = 1/[σ^2^(*F*_o_^2^) + (0.1013*P*)^2^ + 0.2815*P*] where *P* = (*F*_o_^2^ + 2*F*_c_^2^)/3
*S* = 1.05	(Δ/σ)_max_ = 0.001
3361 reflections	Δρ_max_ = 0.97 e Å^−^^3^
138 parameters	Δρ_min_ = −0.56 e Å^−^^3^

### Special details

**Table:**

Geometry. All esds (except the esd in the dihedral angle between two l.s. planes) are estimated using the full covariance matrix. The cell esds are taken into account individually in the estimation of esds in distances, angles and torsion angles; correlations between esds in cell parameters are only used when they are defined by crystal symmetry. An approximate (isotropic) treatment of cell esds is used for estimating esds involving l.s. planes.

### Fractional atomic coordinates and isotropic or equivalent isotropic displacement parameters (Å^2^)

**Table:**

	*x*	*y*	*z*	*U*_iso_*/*U*_eq_	
N1	0.67545 (16)	0.40154 (15)	0.21687 (13)	0.0182 (2)	
H1	0.702337	0.480395	0.131812	0.022*	
N2	0.32976 (16)	0.70943 (15)	0.15397 (13)	0.0187 (2)	
H2	0.447969	0.698634	0.085623	0.022*	
C1	0.82761 (19)	0.26947 (19)	−0.07624 (16)	0.0213 (3)	
H1A	0.844476	0.382168	−0.061678	0.026*	
H1AB	0.942570	0.216566	−0.133336	0.026*	
C2	0.7951 (2)	0.16181 (18)	0.06343 (16)	0.0222 (3)	
H2A	0.911494	0.135809	0.097951	0.027*	
H2AB	0.766041	0.053719	0.048851	0.027*	
C3	0.6416 (2)	0.24003 (18)	0.17554 (15)	0.0202 (3)	
H3A	0.524235	0.264324	0.142544	0.024*	
H3AB	0.628253	0.156392	0.257539	0.024*	
C4	0.8394 (2)	0.3698 (2)	0.2869 (2)	0.0289 (3)	
H4A	0.852000	0.474931	0.316837	0.043*	
H4B	0.951096	0.328712	0.222555	0.043*	
H4C	0.820747	0.285389	0.366416	0.043*	
C5	0.5084 (2)	0.48877 (19)	0.31152 (15)	0.0219 (3)	
H5A	0.541977	0.588041	0.339066	0.026*	
H5AB	0.480340	0.410569	0.395386	0.026*	
C6	0.3326 (2)	0.54741 (19)	0.25240 (16)	0.0220 (3)	
H6A	0.315943	0.456530	0.203864	0.026*	
H6AB	0.226499	0.564160	0.329274	0.026*	
C7	0.3152 (2)	0.8628 (2)	0.22684 (18)	0.0267 (3)	
H7A	0.305431	0.964466	0.161379	0.040*	
H7B	0.424822	0.852681	0.266082	0.040*	
H7C	0.205791	0.870354	0.300087	0.040*	
Cl1	0.21029 (5)	0.17202 (5)	0.52791 (4)	0.02439 (13)	
O1	0.2471 (3)	0.1745 (2)	0.38061 (17)	0.0494 (4)	
O2	0.1539 (3)	0.0175 (3)	0.5935 (3)	0.0669 (6)	
O3	0.3753 (3)	0.1946 (3)	0.5690 (2)	0.0560 (5)	
O4	0.0647 (3)	0.3129 (3)	0.5618 (3)	0.0662 (6)	
Cl2	0.73888 (5)	0.71235 (4)	0.00536 (4)	0.02198 (13)	

### Atomic displacement parameters (Å^2^)

**Table:**

	*U*^11^	*U*^22^	*U*^33^	*U*^12^	*U*^13^	*U*^23^
N1	0.0174 (5)	0.0170 (5)	0.0224 (5)	−0.0036 (4)	−0.0087 (4)	−0.0011 (4)
N2	0.0150 (5)	0.0180 (5)	0.0242 (5)	−0.0023 (4)	−0.0058 (4)	−0.0042 (4)
C1	0.0144 (6)	0.0238 (6)	0.0281 (7)	−0.0034 (5)	−0.0074 (5)	−0.0056 (5)
C2	0.0200 (6)	0.0181 (6)	0.0301 (7)	0.0004 (5)	−0.0105 (5)	−0.0041 (5)
C3	0.0201 (6)	0.0175 (6)	0.0263 (6)	−0.0051 (5)	−0.0095 (5)	−0.0026 (5)
C4	0.0247 (7)	0.0285 (8)	0.0402 (8)	−0.0049 (6)	−0.0196 (6)	−0.0047 (6)
C5	0.0237 (6)	0.0216 (6)	0.0213 (6)	−0.0028 (5)	−0.0066 (5)	−0.0032 (5)
C6	0.0183 (6)	0.0193 (6)	0.0278 (7)	−0.0035 (5)	−0.0043 (5)	−0.0012 (5)
C7	0.0304 (8)	0.0198 (7)	0.0346 (8)	−0.0038 (5)	−0.0133 (6)	−0.0085 (6)
Cl1	0.0237 (2)	0.0222 (2)	0.0294 (2)	−0.00633 (14)	−0.00871 (15)	−0.00105 (14)
O1	0.0545 (10)	0.0648 (11)	0.0346 (7)	−0.0209 (8)	−0.0060 (7)	−0.0131 (7)
O2	0.0720 (13)	0.0481 (10)	0.0887 (15)	−0.0350 (9)	−0.0404 (12)	0.0356 (10)
O3	0.0533 (10)	0.0629 (11)	0.0691 (12)	−0.0267 (9)	−0.0400 (9)	0.0023 (9)
O4	0.0504 (10)	0.0561 (11)	0.0837 (15)	0.0128 (9)	−0.0007 (10)	−0.0290 (10)
Cl2	0.01828 (19)	0.0202 (2)	0.0293 (2)	−0.00692 (13)	−0.00735 (14)	0.00056 (13)

### Geometric parameters (Å, º)

**Table:**

N1—C4	1.5037 (19)	C4—H4A	0.9700
N1—C3	1.5095 (18)	C4—H4B	0.9700
N1—C5	1.5116 (19)	C4—H4C	0.9700
N1—H1	0.9900	C5—C6	1.522 (2)
N2—C7	1.5033 (19)	C5—H5A	0.9800
N2—C6	1.513 (2)	C5—H5AB	0.9800
N2—C1^i^	1.5181 (18)	C6—H6A	0.9800
N2—H2	0.9900	C6—H6AB	0.9800
C1—C2	1.526 (2)	C7—H7A	0.9700
C1—H1A	0.9800	C7—H7B	0.9700
C1—H1AB	0.9800	C7—H7C	0.9700
C2—C3	1.527 (2)	Cl1—O2	1.4180 (17)
C2—H2A	0.9800	Cl1—O1	1.4328 (16)
C2—H2AB	0.9800	Cl1—O4	1.4342 (19)
C3—H3A	0.9800	Cl1—O3	1.4380 (16)
C3—H3AB	0.9800		
			
C4—N1—C3	111.91 (12)	N1—C4—H4A	109.5
C4—N1—C5	108.49 (12)	N1—C4—H4B	109.5
C3—N1—C5	112.37 (11)	H4A—C4—H4B	109.5
C4—N1—H1	108.0	N1—C4—H4C	109.5
C3—N1—H1	108.0	H4A—C4—H4C	109.5
C5—N1—H1	108.0	H4B—C4—H4C	109.5
C7—N2—C6	112.12 (12)	N1—C5—C6	116.19 (12)
C7—N2—C1^i^	111.00 (11)	N1—C5—H5A	108.2
C6—N2—C1^i^	109.81 (11)	C6—C5—H5A	108.2
C7—N2—H2	107.9	N1—C5—H5AB	108.2
C6—N2—H2	107.9	C6—C5—H5AB	108.2
C1^i^—N2—H2	107.9	H5A—C5—H5AB	107.4
N2^i^—C1—C2	113.55 (12)	N2—C6—C5	115.16 (12)
N2^i^—C1—H1A	108.9	N2—C6—H6A	108.5
C2—C1—H1A	108.9	C5—C6—H6A	108.5
N2^i^—C1—H1AB	108.9	N2—C6—H6AB	108.5
C2—C1—H1AB	108.9	C5—C6—H6AB	108.5
H1A—C1—H1AB	107.7	H6A—C6—H6AB	107.5
C1—C2—C3	116.36 (12)	N2—C7—H7A	109.5
C1—C2—H2A	108.2	N2—C7—H7B	109.5
C3—C2—H2A	108.2	H7A—C7—H7B	109.5
C1—C2—H2AB	108.2	N2—C7—H7C	109.5
C3—C2—H2AB	108.2	H7A—C7—H7C	109.5
H2A—C2—H2AB	107.4	H7B—C7—H7C	109.5
N1—C3—C2	114.13 (12)	O2—Cl1—O1	110.75 (13)
N1—C3—H3A	108.7	O2—Cl1—O4	110.14 (15)
C2—C3—H3A	108.7	O1—Cl1—O4	106.85 (14)
N1—C3—H3AB	108.7	O2—Cl1—O3	110.94 (12)
C2—C3—H3AB	108.7	O1—Cl1—O3	108.56 (12)
H3A—C3—H3AB	107.6	O4—Cl1—O3	109.50 (14)
			
N2^i^—C1—C2—C3	−69.05 (16)	C3—N1—C5—C6	−61.62 (16)
C4—N1—C3—C2	−66.31 (16)	C7—N2—C6—C5	−69.95 (16)
C5—N1—C3—C2	171.33 (11)	C1^i^—N2—C6—C5	166.15 (12)
C1—C2—C3—N1	−62.42 (16)	N1—C5—C6—N2	−77.80 (16)
C4—N1—C5—C6	174.11 (13)		

Symmetry code: (i) −*x*+1, −*y*+1, −*z*.

### Hydrogen-bond geometry (Å, º)

**Table:**

*D*—H···*A*	*D*—H	H···*A*	*D*···*A*	*D*—H···*A*
N1—H1···Cl2	0.99	2.13	3.0701 (14)	159
N2—H2···Cl2	0.99	2.17	3.1038 (15)	156
C1—H1*A*···Cl2	0.98	2.77	3.6868 (17)	157
C5—H5*AB*···O3	0.98	2.39	3.351 (3)	167
C3—H3*A*···Cl2^i^	0.98	2.67	3.6274 (17)	164
C3—H3*AB*···O2^ii^	0.98	2.52	3.288 (3)	135
C4—H4*A*···O4^iii^	0.97	2.49	3.429 (3)	164
C4—H4*C*···O2^ii^	0.97	2.39	3.171 (3)	137
C5—H5*A*···O3^iii^	0.98	2.34	3.317 (3)	173
C6—H6*AB*···O4^iv^	0.98	2.31	3.231 (3)	156
C6—H6*A*···Cl2^i^	0.98	2.80	3.7414 (17)	161
C7—H7*B*···O3^iii^	0.97	2.40	3.333 (3)	161

Symmetry codes: (i) −*x*+1, −*y*+1, −*z*; (ii) −*x*+1, −*y*, −*z*+1; (iii) −*x*+1, −*y*+1, −*z*+1; (iv) −*x*, −*y*+1, −*z*+1.
